# Dysregulation of Kisspeptin and Leptin, as Anorexigenic Agents, Plays Role in the Development of Obesity in Postmenopausal Women

**DOI:** 10.1155/2019/1347208

**Published:** 2019-12-01

**Authors:** Andon Hestiantoro, Brilliant P. K. Astuti, Raden Muharam, Gita Pratama, Fiastuti Witjaksono, Budi Wiweko

**Affiliations:** ^1^Reproductive Immunoendocrinology Division, Department of Obstetrics and Gynecology, Faculty of Medicine Universitas Indonesia, Dr. Cipto Mangunkusumo Hospital, Jakarta 10430, Indonesia; ^2^Cluster of Human Reproduction, Fertility and Family Planning, Indonesian Medical Education and Research Institute, Universitas Indonesia, Jakarta 10430, Indonesia; ^3^Department of Nutrition, Faculty of Medicine Universitas Indonesia, Dr. Cipto Mangunkusumo Hospital, Jl. Salemba Raya No. 6, Jakarta 10430, Indonesia

## Abstract

During the menopausal period, women have a higher tendency to develop obesity and any other metabolic syndromes. Dysregulation of leptin and kisspeptin signaling as anorexigenic agents is believed to be the connection between metabolic disorders and altered reproductive function. Therefore, this study aimed at investigating the association between leptin, soluble leptin receptor (sOBR), free leptin index, kisspeptin concentrations, and body mass index (BMI) in postmenopausal women. A cross-sectional study was carried out among 171 postmenopausal women aged 40–75 years from 2017 to 2018. Subjects were assigned into 2 groups according to their BMIs: obese group (84 subjects) and nonobese group (87 subjects). In addition to anthropometric measurement, blood sample was collected from each subject for leptin, sOBR, free leptin index (FLI), and kisspeptin evaluation. Bivariate and correlation analysis discovered that leptin and FLI were positively correlated with BMI, while sOBR and kisspeptin were negatively correlated with BMI. Among those variables, multivariate analysis found that leptin, sOBR, and kisspeptin were independently associated with obesity. Therefore, it can be concluded that higher serum leptin concentration and FLI, as well as lower serum sOBR and kisspeptin concentrations, are significantly associated with obesity in postmenopausal women.

## 1. Introduction

Menopause is a naturally occurring phenomenon among middle-aged women as a consequence of the reproductive aging process. It is a permanent and irreversible termination of the menstrual cycle, which marks the end of reproductive potential. The diagnosis of menopause can only be established after a woman has experienced 12 consecutive months of amenorrhea. During the menopausal period, women go through a significant alteration in reproductive hormone concentration, particularly estrogen, owing to the diminution of ovarian follicular activity [1, 2]. These changes did not only affect the hormonal regulation of hypothalamic-pituitary-gonad (HPG) axis, but also body composition and fat distribution, including weight gain and accumulation of visceral adiposity [3].

Menopause is a widely acknowledged risk factor for obesity in women over the age of 40 years. According to the World Health Organization (WHO), the incidence of obesity in women is highest during the postmenopausal period, between the ages of 55 and 64 years. Hormonal changes following the menopausal period are believed to be the cause of obesity during the postmenopausal period. Estrogen is renowned for its potent anorexigenic effect on energy homeostasis. Hypoestrogenic condition after menopause is proven to induce metabolic dysfunction and decrease basal metabolic rate, hence resulting in higher body mass and body fat percentage, as well as a shift in body fat distribution from gynoid to android [3–5].

The effect of estrogen on energy homeostasis is facilitated by several metabolic signals, and one of the most well-studied is leptin. Leptin is an anorexigenic adipokine, which is secreted by the white adipose tissue in proportion to body fat deposits [6–8]. Estrogen has been shown to increase the expression of leptin genes, implicating that a decrease in estrogen concentration will deliberately result in the reduction of leptin concentration, vice versa [9–11]. Leptin primarily acts at central levels by regulating and maintaining the balance between energy reserves and expenditures. In order to exert its action on the neuroendocrine reproductive system, leptin needs to bind to its receptors: soluble leptin receptor (sOBR). The ratio between leptin and its receptor, which is known as the free leptin index, reflects the condition of leptin resistance and has been proposed as a surrogate marker for metabolic dysfunction [12]. However, recent studies discovered that no leptin receptor was found in GnRH neurons, which raises the question about the presence of potential intermediary neuropeptide facilitating the effect of leptin on the HPG axis [13].

Kisspeptin has arisen recently as a crucial stimulator of GnRH neurons, mediating the effect of leptin on energy metabolism and reproductive axis [14, 15]. Kisspeptin is an endogenous neuropeptide product of Kiss-1 gene, which possesses a fundamental role in the maturation and maintenance of normal reproductive function [16–18]. Many experimental studies conducted on rodents discovered that Kiss-1 system is very susceptible to the changes in energy balance and responds to the stimulatory effect of leptin. These studies discovered that negative energy balance state and its associated metabolic disorders inhibited the hypothalamic expression of Kiss-1 gene. On the other hand, studies on obese or overweight rodents have shown inconsistent results. Some studies found that obesity increased the production and expression of Kiss-1 genes, while other studies discovered no changes or even reduction in Kiss-1 gene expression among obese individuals. It was hypothesized that obesity causes an alteration in Kiss-1 gene expression, depending on the duration and degree of obesity. Therefore, it is well predicted that a condition of disrupted energy homeostasis and metabolic distress would significantly impair Kiss-1 gene expression and alter HPG axis's sensitivity to leptin stimulation [13, 19, 20].

Until recently, there is a general lack of research in the influence of leptin-kisspeptin-GnRH pathways on energy homeostasis in the postmenopausal population. There also has been no general agreement on how obesity affects the function of leptin and kisspeptin upon the reproductive axis. This study therefore set out to assess the association between body mass index (BMI), leptin, and kisspeptin in postmenopausal women.

## 2. Materials and Methods

This was an observational, cross-sectional, single-center study conducted among postmenopausal women in Jakarta. A total of 171 consecutive postmenopausal women were enrolled as study subjects and were included in the analysis. Subject enrollment began in 2017 and completed in 2018 and took place at several Islamic recitation groups in Jakarta. Women were eligible if they were postmenopausal and within the age range of 40 and 75 years. Subjects were excluded if they were suffering from chronic degenerative diseases, such as diabetes, neurological disorders, chronic renal diseases, or pulmonary diseases; active smokers or heavy alcohol drinkers; with surgically or medically induced menopause; or under hormone replacement therapy. Only those women who met the inclusion and exclusion criteria were selected for the study after providing a written consent.

This study planned to enroll 152 women, consisting of 76 subjects in each group. Sample size was calculated to have a confidence level of 90% and 10% differences in the level of precision, and the estimated prevalence of obesity among postmenopausal women was 65%. To account for potential postenrolment dropout, additional 19 subjects were assigned to this study. Therefore, the total number of subjects enrolled in this study was 171.

Individuals were subjected to medical history taking and anthropometric measurements to determine the body mass index (BMI). BMI was categorized according to the World Health Organization (WHO) classification for Asian population, which defines 25 kg/m^2^ as a cutoff point for obesity. Subjects were assigned into two groups according to their BMI: 84 subjects in the obese group and 87 subjects in the nonobese group. A blood sample by 10 mL via a single venepuncture was collected from each subject and sent to a commercial laboratory (Prodia) for leptin, sOBR, kisspeptin, follicle-stimulating hormone (FSH), luteinizing hormone (LH), and estradiol analysis. Free leptin index was calculated as the ratio of leptin to sOBR concentration. Blood samples were collected in heparinized tubes and stored at −80°C in aliquot. Prior to analysis, the samples were thawed at 18 to 25°C.

The quantitative measurement of kisspeptin concentration in plasma was determined using a Human Kisspeptin ELISA Kit (Bioassay Technology Laboratory), with an intra-assay coefficient of variation (CV) of less than 8%, an interassay CV of less than 10%, and a functional sensitivity of 0.5 mIU/L. This assay works according to sandwich ELISA principles, in which a monoclonal antibody specific to Kiss-54 was precoated to the microtiter plates. When the sample was added to the wells, Kiss-54 was bound to its monoclonal antibody (*biotin-conjugated anti-human Kiss-54 antibody*), while the unbound biotin-conjugated anti-human Kiss-54 antibody was washed off. Biotin-conjugated anti-human Kiss-54 antibody was used as a detection antibody. A streptavidin-conjugated horseradish peroxidase (HRP) was added to the wells and incubated, which was later bound to the biotin-conjugated antibody. The unbound streptavidin-HRP conjugate was washed off after the incubation procedures. A tetramethylbenzidine (TMB) solution was added to the microplate wells and reacted to the presence of HRP enzymes, which resulted in the development of specific color. The color intensity of the assay represents the concentration of Kiss-54 within the samples in a reverse proportion. This reaction was terminated by adding a sulfuric acid stop solution. Last, the optical density (OD) value of the microplate wells was measured spectrophotometrically at 450 ± 10 nm wavelength in a microplate reader.

The primary objective of this study was to determine the association between biochemical parameters (such as leptin, sOBR, kisspeptin, and estradiol) and obesity in postmenopausal population. A secondary objective of this study was to evaluate the role of free leptin index (FLI), which reflects leptin resistance condition, in the development of obesity in postmenopausal women. And the tertiary objective of this study was to identify biochemical parameters which were associated independently with obesity in postmenopausal women. The study protocol was approved by the Institutional Review Board of the Faculty of Medicine Universitas Indonesia with an ethical clearance number of 487/UN2.F1/ETIK/V/2017.

Statistical analysis was performed using SPSS (Statistical Package for Social Science) version 22 for Mac. Continuous data are presented as mean ± standard deviation if the variable distribution is normal or as median (minimum-maximum) if the variable distribution is skewed. Normality of the data distribution was defined using Kolmogorov–Smirnov test. Independent *t*-test and Mann–Whitney *U* test were performed to assess between-group differences for normally distributed and nonnormally distributed continuous variables, respectively. Variables resulting in *p* values of 0.05 or less in preliminary bivariate comparisons were included in multivariate logistic regression analysis, with obesity as the outcome measure. Multivariate logistic regression was performed to identify variables which were associated independently with obesity. All statistical analyses were two-sided and a two-tailed *p* value <0.05 was considered significant.

## 3. Results

A total of 171 postmenopausal women consented to participate in this study and were included in the statistical analysis. The study population age ranged from 42 to 74 years, with a mean age of 59.01 ± 6.57 years. According to their body mass indices, subjects were classified into two groups: nonobese (*n* = 87) and obese (*n* = 84). The mean body mass index of the study population was 24.76 ± 3.82 kg/m^2^ (range, 16.44–34.69 kg/m^2^). The baseline characteristics of the study population are presented in Table 1.

The mean age of subjects in the nonobese group was 59.22 ± 6.18 years and in the obese group was 58.79 ± 6.99 years. Obese group comprised younger subjects than the nonobese group, although the difference was not statistically significant (*p*=0.740). Mann–Whitney *U* test showed that leptin concentration was significantly higher in obese subjects compared to the lean counterparts (*p* < 0.001). Free leptin index, which was calculated by dividing leptin by sOBR, was also significantly higher in the obese group compared to the nonobese group (*p* < 0.001). On the other hand, sOBR and kisspeptin serum concentrations were significantly lower in obese subjects compared to the lean ones (*p*=0.001 and *p* < 0.001, respectively). FSH, LH, and estradiol concentration, as well as FSH to estradiol ratio, showed no significant differences between obese and nonobese population (Table 2).

**Figure 1:**
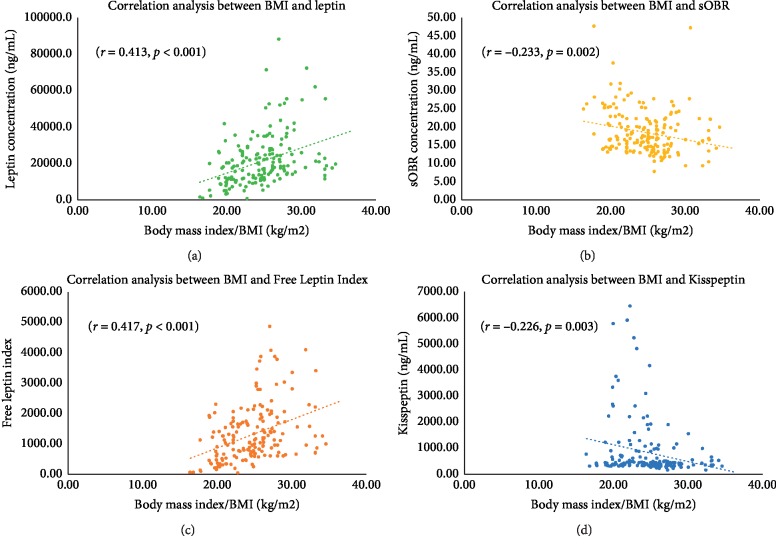
The scatter plot of correlation analysis between (a) body mass index and leptin, (b) body mass index and sOBR, (c) body mass index and FLI, and (d) body mass index and kisspeptin. According to the correlation analysis, both leptin and FLI were positively correlated with FLI, while sOBR and kisspeptin were negatively correlated with FLI.

Spearman correlation analysis indicated that leptin and free leptin index were positively correlated with BMI, while sOBR and kisspeptin were negatively correlated with BMI. Both leptin (*r* = 0.413, *p* < 0.001) and free leptin index (*r* = 0.417, *p* < 0.001) had strong degrees of association with BMI. On the contrary, sOBR (*r* = −0.233, *p*=0.002) and kisspeptin (*r* = −0.226, *p*=0.003) had weak degrees of association with BMI (Figure 1).

Multivariate linear regression analysis revealed that leptin was independently and positively associated with body mass index, while sOBR and kisspeptin levels were independently and negatively associated with body mass index in postmenopausal population (Table 3).

## 4. Discussion

Obesity is the most prevalent form of malnutrition, in both developed and developing countries in the world. It has been recognized as a worldwide epidemic in the past few decades, affecting approximately one-third of the world's population. Gender differences in the prevalence of obesity have been observed between races and countries, where it was found that women were 1.5 times more likely to become obese compared to men [21]. As previously stated above, estrogen plays an important role in the regulation of energy homeostasis through its strong influences on glucose and fat metabolism, as well as the control of appetite. Estrogen has the ability to regulate body fat distribution and suppress appetite by inhibiting the action of orexigenic neuropeptides in the hypothalamus. Ovarian hormone deficiency during the menopausal stage is thought to trigger hyperphagia and induce a disruption in energy homeostasis, resulting in the development of obesity and other metabolic disorders [3, 4, 22–25].

Estrogen helps regulate energy homeostasis by influencing both hypothalamus centrally and adipose tissue peripherally. The primary actions of estrogen on the hypothalamus are exerted through the binding of its molecules to estrogen receptors, ER-*α* and ER-*β*. Estrogen receptors are prevalently expressed in the infundibular (arcuate) nucleus (INF/ARC), anteroventral periventricular nucleus (AVPV), medial preoptic area, and ventrolateral portion of ventromedial nucleus (VMN). These receptors are expressed in a distinct pattern: ER-*α* predominates in the arcuate and ventromedial portion, while ER-*β* predominates in the paraventricular portion of the hypothalamus. Even though both receptors are capable of mediating energy homeostasis function, the influence of estrogen on metabolic control is mainly mediated by ER-*α*. The binding of estrogen to ER-*α* in INF/ARC amplifies the excitatory input of proopiomelanocortin (POMC) neurons, which in turn causes a repression in calorie intake. In accordance with this, the binding of estrogen to ER-*α* in VMN also activates the transcription of steroidogenic factor-1 (SF-1), which plays an important role in the regulation of energy expenditure and body fat composition. Together with its role in lipid metabolism and adiposity regulation in peripheral tissue, estrogen acts as a powerful anorexigenic agent of the human body. The results obtained from our comparative bivariate analysis showed that both obese and nonobese groups had relatively equal estradiol concentration, suggesting the potential influence of other factors on energy homeostasis, such as lifestyle and daily dietary intake [26, 27].

The median BMI of our study population was 24.76 ± 3.82 kg/m2, which was classified as overweight according to WHO Asia Pacific BMI criteria. Although it was not considered as obese yet, it did confirm the premises which stated that postmenopausal women tend to have higher body mass compared to premenopausal women. Epidemiological data from the United States have shown that the incidence of obesity among women between the ages of 40 and 65 years was 65%. This incidence would reach its peak after the age of 65 years, where it was documented that the incidence of obesity reached 74% [24, 25].

The current study found that leptin, sOBR, and FLI were significantly correlated with body mass index in postmenopausal women. Increases in leptin concentration and FLI were observed in obese women, while sOBR concentration was found to be lower. These findings fit the theory which stated that the secretion of leptin is commensurable to the size of body fat. Leptin is a satiety agent that is synthesized as a sign of energy adequacy [9–11]. In the same vein with estrogen, leptin primarily acts on INF/ARC by modulating the expression of several orexigenic neuropeptides, such as neuropeptide-Y (NPY), and anorexigenic neuropeptides, such as POMC and cocaine and amphetamine-regulated transcript (CART) [13, 28]. In the human circulatory system, leptin binds to sOBR in order to maintain its bioavailability and slow down its clearance. sOBR is an important contributor in the enhancement of leptin functions. A number of cross-sectional studies have found that sOBR was conversely associated with adiposity and obesity, which corroborates with the finding of this study [29–31].

Sexual dimorphism in leptin-adiposity relationship has been observed between men and women, suggesting the importance of sexual hormones in leptin regulation [32]. Estrogen is proven to intensify the sympathoexcitatory effect of leptin, resulting in higher leptin concentration [31]. A large and growing body of literature has investigated the impact of long-term estrogen deprivation and the benefit of hormone replacement therapy on leptin levels. Widely divergent results have been reported among these studies. Some studies found that hypoestrogenic condition, such as found in postmenopausal women, causes a significant reduction in leptin concentration. Among postmenopausal women, those who were obese had significantly higher leptin concentration compared to the nonobese counterpart [33]. These studies implicate the significance of menopausal status as a determinant of leptin concentration. In contrast to the previously mentioned results, some other studies found that the change in leptin concentration was independent of menopausal status. BMI was found to be the only important determinant of leptin in women [32].

Even though leptin presents in a strikingly high concentration, marked hyperphagia is still observed among the obese population. Some studies have shown that obese people required up to 20–30 times higher leptin levels in order to exert its inhibitory effect on food intake and produce significant weight losses. This condition indicates the presence of leptin resistance, which significantly attenuates leptin's regulatory properties on energy homeostasis. Moreover, the expression of leptin genes increases as someone gets older which supposedly results in higher leptin concentration, denoting to the possibility of age-associated leptin resistance. Leptin resistance is clinically defined as free leptin index, a ratio between leptin and sOBR. This study found that FLI was moderately correlated with body mass index in a positive manner. The present findings seem to be consistent with the findings of other studies, which discovered that impairment in leptin signaling was commonly found among people with diet-induced obesity [34–36].

Despite its prominent function in the regulation of energy homeostasis and reproductive function in the hypothalamus, no leptin receptor was ever observed in GnRH. Intermediate pathways by means of several neuropeptides, such as NPY, CART, and POMC, were proposed as a link between leptin and GnRH neurons. Recently, kisspeptin has been studied extensively as a possible interneuronal link between leptin and GnRH neurons. However, most of these studies were performed among undernourished women. Not much study has been conducted to evaluate the association between kisspeptin and metabolic control among obese women. Some of the studies conducted on rats discovered that obesity caused a disruption in kisspeptin signaling and a reduction in kisspeptin's sensitivity to sex hormone stimulation. These studies corroborate with the results of our study, which discovered a negative correlation between BMI and kisspeptin concentration. On the other hand, a study on male rats found that high-fat meal caused a transient increase in Kiss-1 mRNA expression. Another study performed on Saudi women also found no significant difference in kisspeptin concentration between normal and overweight subjects. In accordance with the aforementioned findings among these Saudi women, a study performed by Çelik on 83 Turkish women also found that kisspeptin concentration was not influenced by changes in body mass indices or menopausal status. A possible explanation for these rather contradictory results may be the differences in the characteristic of the study population. Other factors or possible confounders such as daily nutrient intake, physical activity, and body composition were not assessed extensively in this study, which might influence the changes in kisspeptin concentration [13, 19, 28, 37, 38].

Just like leptin, kisspeptin neurons are also expressed mainly in INF/ARC and AVPV. The regulation of kisspeptin neurons is partly influenced by leptin and sex hormones, where an increase in leptin and sex hormone concentration is associated with higher kisspeptin concentration. An experimental study conducted on mice showed that dysfunctional leptin signaling, such as leptin resistance or leptin deficiency, caused a remarkable reduction in Kiss-1 gene expression. This reduction was also observed among people with diet-induced obesity [13, 19, 28, 37]. Although not specifically presented in the Result section, the authors observed a statistically significant negative correlation between FLI and kisspeptin (*r* = −0.154, 0.045).

It was also interesting to note that even though there were only slight alterations in body mass index classification, this study found strong associations between leptin, free leptin index, and body mass index. One of the many functions of leptin was to notify the brain about the levels of stored fat among one's body. It was important to note that the changes in body fat distribution and concentration were the key factors in triggering leptin's action. Body mass index itself was not reflective of the levels of stored fat in someone's body. Changes in body weight, which further cause changes in body mass indices, might be caused by changes in water concentration, fat concentration, or muscle mass. Therefore, these strong associations between leptin, free leptin index, and BMI might be caused by changes in fat distribution or concentration, which were not assessed thoroughly in this study [39].

It is hypothesized that hormonal changes commonly found in the postmenopausal population, including the hormones responsible for energy homeostasis, were the consequences of the aging process of hypothalamus. The role of the hypothalamus in energy homeostasis was mediated through the presence of several neurons, such as the orexigenic agouti-related peptide (AgRP) and NPY, as well as the anorexigenic POMC. Long isoform of the leptin receptor (LERP-B), the receptors which are responsible for mediating the effect of leptin on the hypothalamus, are expressed abundantly among these 3 neurons. The activities of these neurons decreased with age and their responses to stimuli were blunted through the deregulation of mTOR, increased activation of I*κ*B kinase-*β* and nuclear factor *κ*B, loss of hypothalamic neural stem cells (htNSC), and reduced autophagy of POMC neurons [40].

htNSC was accountable for the expression of certain phenotypes, such as the activation of signal transducer and activator of transcription 3 (STAT3) by leptin as a long-term regulator of energy balance. The loss of htNSC due to aging was presumed to be one of the underlying mechanisms of leptin resistance. In addition to its altered function in the regulation of energy metabolism, these changes also downregulated the expression and transcription of GnRH. The activities of GnRH pulse generator, such as kisspeptin, also decreased in the aging population due to the diminution of the KNDy neurons which constitute the positive regulatory feedback loop. As a result of the aging process, the sensitivity of the hypothalamus to regulatory feedback decreased; hence, greater feedback stimulus was necessary to preserve its function. A study on hemicastrated rodents discovered that older rodents required a higher dose of administered estrogen compared to the younger ones in order to suppress the production of gonadotropins, which further confirms that the sensitivity of hypothalamus to estrogen feedback decreased with age. This theory corroborates with the findings of our study, which discovered no significant differences in FSH and estrogen concentrations between the two study groups. These findings indicated that estrogen feedback to gonadotropin did not differ between obese and nonobese populations. Notwithstanding this almost identical feedback, the concentration of kisspeptin differed significantly between the two study groups, which implicates the existence of other factors in charge of kisspeptin regulation [40–42].

This study, however, had a number of limitations which need to be considered. First, the number of samples included in this study was relatively small. Therefore, the findings of this study might not be representative of the overall postmenopausal population. In addition to the number of sample size, the observational nature of this cross-sectional study made it impossible for researchers to determine the causal inferences between the study variables. Therefore, future studies with a larger sample size and adequate statistical power need to be done in order to validate the findings of this study. Second, there are several vital anthropometric and body composition measurements which were not assessed in this study, such as waist circumference, hip circumference, and body fat percentage. This study also did not assess the daily nutrient intake of the study subjects. As previously stated, changes in body fat distribution might influence the changes in hormonal as well as neuropeptide concentrations in postmenopausal women. By not assessing these factors, this study possibly missed some pivotal points in explaining how obesity affects hormonal changes among the postmenopausal population.

## 5. Conclusions

Taken together, these results suggest that higher serum leptin concentration and free leptin index, as well as lower serum sOBR and kisspeptin concentrations, are significantly associated with obesity in postmenopausal women. The conclusion of the causal relationship between leptin, sOBR, kisspeptin, and nutritional status in postmenopausal women cannot be drawn in the present study. Considerably more research studies on this topic need to be carried out before the association between these biochemical parameters and nutritional status is more clearly elucidated.

## Figures and Tables

**Table 1:** The characteristics of the overall study population.

Characteristic	Value (*n* = 171)
Age (years)	59 (42–74)
Body height (meter)	1.53 (1.40–1.68)
Body weight (kg)	59 (37–79)
Body mass index (BMI) (kg/m^2^)	24.76 ± 3.82
Leptin levels (ng/mL)	18230 (390.00–87717.00)
sOBR levels (ng/mL)	16.80 (7.58–47.47)
Free leptin index	1102.66 (13.40–4832.89)
Kisspeptin levels (ng/mL)	396.72 (126.16–6430.92)
Follicle-stimulating hormone/FSH levels (mIU/mL)	37.69 (10.41–177.59)
Luteinizing hormone/LH levels (mIU/mL)	66.25 (6.31–191.90)
Estradiol levels (pg/dL)	13.50 (11.80–113.80)

**Table 2:** Comparative analysis between obese and nonobese population.

Variables	Nonobese (*n* = 87)	Obese (*n* = 84)	*p* value
Age (years)	59 (43–74)	58 (42–73)	0.667
Body mass index (BMI) (kg/m^2^)	21.80 ± 2.08	27.83 ± 2.58	
Leptin levels (ng/mL)	13114 (390–41307.10)	21817.30 (7170.60–87717)	<0.001
sOBR levels (ng/mL)	17.83 (12.69–47.47)	16.43 (7.58–46.95)	0.001
Free leptin index	768.11 (13.40–2282.98)	1377.84 (537.53–4832.89)	<0.001
Kisspeptin levels (ng/mL)	435.80 (275.49–6430.92)	353.74 (126.16–1893.25)	<0.001
FSH levels (mIU/mL)	37.44 (10.41–126.38)	37.79 (14.71–177.59)	0.897
LH levels (mIU/mL)	61.06 (23.74–149.19)	69.34 (6.31–191.90)	0.420
Estradiol levels (pg/mL)	13.60 (11.80–113.80)	13.25 (11.80–68.50)	0.814
FSH to estradiol ratio (mIU/pg)	2.51 (0.27–10.71)	2.28 (0.28–15.05)	0.734

**Table 3:** The results of the multivariate linear regression model with body mass index as outcome and biochemical parameters as independent covariates.

Variables	Unstandardized B	*t*	*p* value	95% CI for ExpB	
				Lower	Upper
Leptin	0.000	2.377	0.019	0.000	0.000
sOBR	−0.165	−2.350	0.020	−0.304	−0.026
FLI	−0.001	−0.921	0.358	−0.003	0.001
Kisspeptin	−0.001	−2.481	0.014	−0.001	0.000

## Data Availability

The data used to support the findings of this study are available from the corresponding author upon request.
